# Same‐day antiretroviral therapy (ART) initiation in pregnancy is not associated with viral suppression or engagement in care: A cohort study

**DOI:** 10.1002/jia2.25133

**Published:** 2018-06-22

**Authors:** Nontokozo Langwenya, Tamsin K Phillips, Kirsty Brittain, Allison Zerbe, Elaine J Abrams, Landon Myer

**Affiliations:** ^1^ Division of Epidemiology and Biostatistics School of Public Health and Family Medicine University of Cape Town Cape Town South Africa; ^2^ Centre for Infectious Diseases Epidemiology & Research University of Cape Town Cape Town South Africa; ^3^ ICAP at Columbia University Mailman School of Public Health New York New York; ^4^ Columbia University College of Physicians & Surgeons New York New York

**Keywords:** viral suppression, antiretroviral initiation, Option B+, pregnancy, patient preparation, patient counselling, Prevention of Mother to Child Transmission (PMTCT), retention

## Abstract

**Introduction:**

Many prevention of mother‐to‐child HIV transmission programmes across Africa initiate HIV‐infected (HIV positive) pregnant women on lifelong antiretroviral therapy (ART) on the first day of antenatal care (“same‐day” initiation). However, there are concerns that same‐day initiation may limit patient preparation before starting ART and contribute to subsequent non‐adherence, disengagement from care and raised viral load. We examined if same‐day initiation was associated with viral suppression and engagement in care during pregnancy.

**Methods:**

Consecutive ART‐eligible pregnant women making their first antenatal care (ANC) visit at a primary care facility in Cape Town, South Africa were enrolled into a prospective cohort between March 2013 and June 2014. Before July 2013, ART eligibility was based on CD4 cell count ≤350 cells/μL (“Option A”), with a 1 to 2 week delay from the first ANC visit to ART initiation for patient preparation; thereafter all women were eligible regardless of CD4 cell count (“Option B+”) and offered ART on the same day as first ANC visit. Women were followed with viral load testing conducted separately from routine ART services, and engagement in ART services was measured using routinely collected clinic, pharmacy and laboratory records through 12 months postpartum.

**Results:**

Among 628 HIV‐positive women (median age, 28 years; median gestation at ART start, 21 weeks; 55% newly diagnosed with HIV), 73% initiated ART same‐day; this proportion was higher under Option B+ *versus* Option A (85% *vs*. 20%). Levels of viral suppression (viral load <50 copies/mL) at delivery (74% *vs*. 82%) and 12 months postpartum (74% *vs*. 71%) were similar under same‐day *versus* delayed initiation respectively. Findings were consistent when viral suppression was defined at <1000 copies/mL, after adjustment for demographic/clinical measures and across subgroups of age, CD4 and timing of HIV diagnosis. Time to first viral rebound following initial suppression did not differ by timing of ART initiation nor did engagement in care through 12 months postpartum (same‐day = 73%, delayed = 73%, *p* = 0.910).

**Conclusions:**

These data suggest that same‐day ART initiation during pregnancy is not associated with lower levels of engagement in care or viral suppression through 12 months post‐delivery in this setting, providing reassurance to ART programmes implementing Option B+.

## 1 Introduction

Use of triple‐drug antiretroviral therapy (ART) reduces the risk of mother‐to‐child transmission (MTCT) by suppressing maternal viral load (VL) during pregnancy, labour and breastfeeding 1, 2, 3. Each additional week on ART prior to delivery has been shown to reduce the risk of MTCT by up to 8%, thus maximizing ART exposure through rapid ART initiation during antenatal care (ANC) is a priority for all prevention of mother‐to‐child transmission (PMTCT) programmes 4, 5, 6, 7. Historically, CD4 cell count as a marker of advanced disease was required to determine treatment eligibility among pregnant and breastfeeding women 8, 9. ART‐eligible pregnant women were referred for patient education and completion of pre‐treatment counselling prior to ART initiation 10. The need for CD4 cell count enumeration and pre‐ART counselling contributed to significant delays in ART initiation during pregnancy, particularly in Sub‐Saharan Africa where there is limited access to laboratory services and great variation in materials and delivery methods for patient preparation 5, 11.

In the last decade, several approaches have been adopted to facilitate rapid ART initiation during pregnancy. Use of point‐of‐care tests for CD4 cell enumeration and mobile text messaging for delivery of CD4 count results to patients have decreased delays associated with CD4 enumeration 12, 13, 14. In parallel, there have been major advances in integrating ART provision into ANC, reducing delays associated with the referral of women between ANC and ART clinics 15. More recently, many countries have shifted to providing lifelong ART to all HIV‐infected pregnant and breastfeeding women regardless of CD4 count or clinical indication (the World Health Organization's (WHO) “Option B+”) 8. Option B+ eliminates the need for CD4 enumeration and allows HIV‐infected pregnant women to start lifelong ART on the day of their first ANC visit – “same‐day” ART initiation 16, 17, 18.

Same‐day ART initiation in pregnancy has potential advantages as well as limitations. By maximizing the duration of ART during pregnancy, same‐day initiation may contribute to an increased likelihood of achieving viral suppression (VS) prior to delivery and reduced HIV transmission 19. Same‐day initiation may also reduce attrition in the ART initiation “cascade” by increasing the proportion of HIV‐infected pregnant women who start ART 20. However, same‐day initiation also reduces the opportunity for pre‐ART patient education and counselling, which are traditionally a core component of ART services 21. Pre‐treatment counselling is thought to play an important role in preventing patient disengagement and non‐adherence following ART initiation, particularly in pregnant women whose motivation for starting ART is not only for personal health, but often primarily to protect their HIV‐exposed infant from MTCT 22, 23.

Data are conflicting on the impact of same‐day ART initiation on maternal health. In Malawi, pregnant women who were offered ART same‐day were five times less likely to return for an ART follow‐up visit compared to women initiating ART due to CD4 ≤ 350 cells/μL, with authors citing limited understanding of the initial ART education session 24. In contrast, our research group found that, under CD4 based eligibility, delaying ART initiation for patient preparation among HIV‐infected pregnant women was not associated with improved maternal outcomes (engagement in care and VS) at 12 months after initiation in South Africa 25. Similarly in Uganda, comparable VS rates were observed in pregnant women initiating ART same‐day under Option B+ and those starting ART based on CD4‐based criterion (82% *vs*. 80%) 26. Given the paucity of data on this topic, we examined whether same‐day initiation was associated with engagement in care and VS in a cohort of HIV‐infected women initiating ART during pregnancy in Cape Town, South Africa.

## 2 Methods

This is a secondary analysis of a larger study evaluating strategies to optimize ART services for maternal and child health (MCH‐ART; Clinical Trials‐gov NCT01933477) in Cape Town, South Africa 27. Between March 2013 and June 2014, we enrolled consecutive HIV‐infected pregnant women who were ART‐eligible, 18 years or older and attending their first ANC visit at a large primary healthcare facility. All women were followed from their first ANC visit to six weeks post postpartum, with VL testing and study interviews conducted separately from ART services. All study viral loads were batched and results were only available at the end of the study period, with the exception of delivery and 12 months (12 m) postpartum viral loads where women with VL > 1000 copies/mL being referred immediately to routine services for counselling. If reporting to be breastfeeding at first postpartum visit, women attended additional study visits with VL testing until 12 m postpartum. To assess engagement in ART services, all women regardless of breastfeeding practices had their routinely collected clinic, pharmacy and laboratory healthcare records abstracted through 12 m postpartum. All women completed written informed consent for study participation and ethical approval was provided by the Human Research Ethics Committee of the University of Cape Town and the Institutional Review Board of Columbia University Medical Centre.

### 2.1 ART services

Before July 2013, pregnant women were identified as ART‐eligible according to WHO's Option A guidelines based on CD4 cell count ≤350 cells/μL and/or WHO clinical stage III/IV. Under this approach, ART‐eligible pregnant women received at least two pre‐therapy counselling sessions: the first at one to two weeks before initiation and the second on the day of ART dispensing. After July 2013, lifelong ART was provided at first ANC visit irrespective of CD4 cell count or WHO clinical stage, as per WHO's Option B+, with all women receiving one pre‐therapy counselling session on the day of their first ANC visit. Under both Option A and Option B+, counselling was provided by trained counsellors during a 15‐minute group session covering dose schedules, treatment side effects, adherence and prevention of drug resistance. Further one‐on‐one counselling was provided at ART dispensing and women received ongoing counselling and support at subsequent ART refill visits. Throughout the study period, women received a single fixed‐dose combination of tenofovir, emtricitabine and efavirenz (TDF + FTC + EFV) to be taken once daily, with a month's supply of treatment being provided for the first four months of treatment 28. Thereafter, women received one to two months’ supply of treatment at the discretion of the provider.

### 2.2 Data collection

During pregnancy, study visits were synchronized with routine ANC visits, allowing for a maximum of three study visits depending on gestation at first ANC visit. Women were scheduled to return two weeks after their first ANC visit, at 36 weeks gestation and within seven days of delivery. Postpartum study visits were scheduled at six weeks and 3, 6, 9 and 12 months postpartum. VL testing was done at first ANC visit and at each subsequent study visit. Data on demographic characteristics, clinical history, ART initiation and ART use were collected through face‐to‐face interviews conducted in the local language, isiXhosa. Clinical data were abstracted from paper and electronic participant medical records.

### 2.3 Analysis

We defined ART delay as days since first ANC visit to ART initiation, with date of ART initiation being self‐reported by participants and medical records were reviewed to assess consistency in reporting and date of first ART dispensing. Delay to ART initiation after first ANC visit was analysed as a proxy for the time available for patient preparation, with all women receiving at least one pre‐therapy counselling session on the day of ART initiation. Same‐day ART initiation was defined as starting ART within 48 hours of first ANC visit. The outcomes of interest included (i) VS at delivery, (ii) time to first unsuppressed VL following initial suppression and (iii) engagement in care at 12 m postpartum. Suppressed VL was defined as VL < 50 copies/mL and VL data used to assess suppression at delivery was restricted to data collected ±7 days of delivery. In a sensitivity analysis, we redefined VS as VL < 1000 copies/mL and included VL testing data up to six weeks post‐delivery. We found that variations in definitions and inclusion period did not influence results appreciably, and thus present the findings using VS as VL < 50 copies/mL. Participants were defined as engaged in care at 12 m postpartum if having a documented clinic, pharmacy or laboratory records at any ART facility within the country between 9 and 18 months postpartum. Participants who only had evidence of acute inpatient care unrelated to HIV were classified as not engaged in care.

Data were analysed using Stata Version 13 (Stata Corporation, College Station, USA). Bivariate analysis used Wilcoxon rank‐sum and Kruskal–Wallis tests for continuous variables and Chi‐squared and Fisher's exact tests for categorical variables. We investigated the association between delay to ART initiation and VS at delivery using logistic regression, reporting relative odds of achieving VS with 95% confidence intervals (CI). Sensitivity analyses were conducted to determine if the odds of VS changed significantly in the adjusted model when restricted to women with CD4 ≤ 350 cells/μL or initiating ART under Option B+. Variables were included in the model if known to be a confounder and/or if independently associated with the outcome during bivariate analysis. Model building used likelihood ratio test and the Akiake Information Criterion (AIC). We used survival analysis methods to analyse time to first unsuppressed VL following initial suppression; this was restricted to women who achieved VS and had at least one VL measure after initial VS. We censored women at the date at which VL ≥ 50 copies/mL was later recorded or at their last study visit if their VL was still suppressed or lost to follow‐up. Lastly, we investigated the association between delay to ART initiation and engagement in care at 12 m postpartum using logistic regression.

## 3 Results

### 3.1 Characteristics at the time of ART initiation

A total of 642 ART‐eligible pregnant women presented for ANC and were eligible for study participation, of whom 628 were enrolled. Of these, 81% (n = 508) initiated ART under Option B+. The baseline characteristics of the cohort at first ANC visit and by timing of ART initiation are shown in Table 1. The median age and gestation was 28 years (IQR = 24 to 32) and 21 weeks (IQR = 16 to 26) respectively. The majority of women had completed primary education (96%), were living in informal housing (53%) and were newly diagnosed as HIV‐infected during the current pregnancy (55%). Among those diagnosed with HIV prior to the current pregnancy (n = 283), more than half (52%) were diagnosed during a previous pregnancy of which 86% reported exposure to ARV prophylaxis for PMTCT during that pregnancy. The median CD4 cell count at first ANC visit was 343 cells/μL (IQR = 235 to 506), with 52% of the cohort having a CD4 ≤ 350 cells/μL.

**Table 1:** Demographic, obstetric and clinical characteristics of 628 women stratified by timing of ART initiation

	Total n = 628	Same‐day initiation n = 456 (73%)	Delayed‐initiation n = 172 (27%)	*p* value
**Demographics**				
Age (years)^a^	28 (24 to 32)	28 (24 to 32)	28 (25 to 32)	0.745
Level of education				
Primary school	26 (4%)	16 (4%)	10 (6%)	
High school & above	601 (96%)	439 (96%)	162 (94%)	0.198
Occupation				
Not scholar/employed	389 (62%)	289 (64%)	100 (58%)	
Scholar/Employed	238 (38%)	166 (36%)	72 (42%)	0.216
Housing				
Informal (shack)	333 (53%)	236 (52%)	97 (56%)	
Formal (house/hostel)	294 (47%)	219 (48%)	75 (44%)	0.311
Relationship status				
Single/Not married	477 (76%)	349 (77%)	128 (74%)	
Married	150 (24%)	106 (23%)	44 (26%)	0.550
Gravidity				
Nulli /Prima	374 (60%)	265 (58%)	109 (63%)	
Multi	253 (40%)	190 (42%)	63 (37%)	0.243
Timing of HIV diagnosis				
Current pregnancy	344 (55%)	260 (57%)	84 (49%)	
Before current pregnancy	283 (45%)	195 (43%)	88 (51%)	0.062
**Among those diagnosed with HIV before pregnancy (n = 283)**				
Years since HIV diagnosis				
0 to 5 years	183 (65%)	125 (64%)	58 (66%)	0.769
6 years or more	100 (35%)	70 (36%)	30 (34%)	
Reason for testing at HIV diagnosis				
Pregnancy (PMTCT)	146 (52%)	105 (54%)	41 (47%)	
VCT	76 (27%)	54 (28%)	22 (25%0	
Medical (TB/STI/sick)	61 (22%)	36 (18%)	25 (28%)	0.168
Disclosed HIV status to anyone				
No	26 (9%)	21 (11%)	5 (6%)	
Yes	257 (91%)	174 (89%)	83 (94%)	0.170
**Clinical**				
Median Gestation^b^ (weeks)	21 (16 to 26)	22 (17 to 28)	17 (11 to 23)	<0.0001
Median VL (log10 copies/mL)	3.99 (3.38 to 4.64)	3.935 (3.27 to 4.56)	4.131 (3.53 to 4.70)	0.0133
VL < 50 copies/mL	23 (4%)	20 (4%)	3 (2%)	0.153
VL < 1000 copies/mL	100 (16%)	80 (18%)	20 (12%)	0.071
Median CD4 count^c^ (cells/μL)	343 (235 to 506)	378 (245 to 545)	283 (214 to 375)	<0.0001
≤350 cells/μL	314 (52%)	200 (45%)	114 (68%)	<0.001
PMTCT Option A	120 (19%)	23 (5%)	97 (56%)	
Option B+	508 (81%)	433 (95%)	75 (44%)	<0.001

Used Wilcoxon rank sum test, Fisher Exact test and chi‐squared test.

a Missing data for 2 participants.

b Missing data for 4 participants.

c Missing data for 18 participants.

### 3.2 Timing of initiation of antiretroviral therapy

Overall, 73% of women (n = 456) initiated ART within 48 hours of first ANC visit. Among 172 women (27%) who delayed ART initiation beyond 48 hours, the median delay to ART initiation was seven days (IQR = 7 to 32; range 3 to 133) (Figure S1). There were no significant differences in demographic characteristics between the two groups (Table 1). However, women who started ART same‐day presented for ANC at a later gestation compared to women who delayed ART initiation (*p* < 0.001) and had a higher median CD4 cell count (*p *< 0.001) at first ANC visit. The majority of women who initiated ART same‐day were enrolled under Option B+ (95% *vs*. 5%). In the cohort, seven women experienced delay to ART initiation associated with tuberculosis (TB) diagnosis in pregnancy.

### 3.3 Viral suppression at delivery

Of the 628 women enrolled, 68% (n = 428) had VL data within ±7 days of delivery, this proportion did not differ by timing of ART initiation (same day = 69% *vs*. delayed 66%, *p* = 0.417). However, women with VL testing at delivery were older and had presented for ANC at later gestation compared to those with no VL testing (median age = 28 years *vs*. 27 years, median gestation = 21 weeks *vs*. 20 weeks). Of the 428 women with VL at delivery, 23% (n = 100) had a VL ≥ 50 copies/mL (median = 187; IQR = 96 to 1061). Table 2 shows baseline characteristics stratified by VS at delivery. There was no difference in VS at delivery by timing of ART initiation (71% *vs*. 80% among same‐day initiators; *p* = 0.097). Compared to women with unsuppressed VL, women with suppressed VL entered ANC earlier, had a higher CD4 cell count and lower VL at first ANC visit (all *p *< 0.001). There were no differences in demographic characteristics and HIV history between women with suppressed *versus* unsuppressed VL at delivery; median age (28 years *vs*. 28 years), proportion married (25% *vs*. 20%) and proportion diagnosed HIV positive during current pregnancy (44% *vs*. 42%). These findings persisted when the seven women with delayed ART initiation due to TB were excluded from the analysis.

**Table 2:** Demographic, obstetric and clinical characteristics of women with viral load data at delivery stratified by viral suppression (VS < 50 copies/mL)

	Total n = 428	Suppressed n = 328 (77%)	Not Suppressed n = 100 (23%)	*p* value
**Demographics**				
Age (years)^a^	28 (25 to 32)	28 (25 to 32.5)	28 (25 to 31)	0.504
Level of education				
Primary school	19 (4%)	15 (5%)	4 (4%)	
High school& above	409 (96%)	313 (95%)	96 (96%)	0.808
Occupation				
Not scholar/employed	259 (61%)	198 (60%)	61 (61%)	
Scholar/Employed	169 (39%)	130 (40%)	39 (39%)	0.910
Housing				
Informal (shack)	223 (52%)	172 (52%)	51 (51%)	
Formal (house/hostel)	205 (48%)	156 (48%)	49 (49%)	0.801
Relationship status				
Single/Not married	327 (76%)	247 (75%)	80 (80%)	
Married	101 (43%)	81 (25%)	20 (20%)	0.333
Gravidity				
Nulli /Prima	245 (57%)	183 (56%)	62 (62%)	
Multi	183 (43%)	145 (44%)	38 (38%)	0.272
Timing of HIV diagnosis				
Current pregnancy	243 (57%)	185 (56%)	58 (58%)	
Before current pregnancy	185 (43%)	143 (44%)	42 (42%)	0.778
**Among those previously diagnosed (n** = **186)**				
Years since HIV diagnosis				
0 to 5 years	115 (62%)	83 (58%)	32 (74%)	
6 years or more	71 (38%)	60 (42%)	11 (26%)	0.053
Reason for testing at HIV diagnosis				
Pregnancy (PMTCT)	91 (49%)	75 (52%)	16 (38%)	
VCT	51 (28%)	38 (27%)	13 (31%)	
Medical (TB/STI/sick)	43 (23%)	30 (21%)	13 (31%)	0.226
Disclosed HIV status to anyone				
No	21 (11%)	17 (12%)	4 (10%)	
Yes	164 (89%)	126 (88%)	38 (90%)	0.671
**Clinical**				
Median Gestation^b^ (weeks)	21 (16 to 27)	20 (15 to 25)	26 (22 to 32)	<0.001
Median Viral load (log10 copies/mL)	4.008 (3.38 to 4.63)	3.856 (3.18 to 4.45)	4.573 (4.01 to 4.95)	<0.001
Median CD4 count^c^ (cells/μL)	338 (235 to 481)	353 (252 to 514.5)	273 (173 to 403)	<0.001
≤350 cells/μL	219 (53%)	157 (49%)	62 (65%)	0.006
PMTCT Option A	80 (19%)	65 (20%)	15 (15%)	
Option B+	348 (82%)	263 (80%)	85 (85%)	0.279
Median mths on ART at delivery	4.050 (2.64 to 5.24)	4.400 (3.28 to 5.44)	2.628 (1.48 to 3.91)	<0.001
Timing ART initiation				
Delayed	113 (26%)	93 (28%)	20 (20%)	
Same‐day (0 to 2 days)	315 (74%)	235 (71%)	80 (80%)	0.097

Use Wilcoxon ranksum test and chi‐squared test.

a Missing data for 2 participants.

b Missing data for 4 participants.

c Missing data for 18 participants.

Predictors of VS at delivery are presented in Table 3. After adjusting for demographic and clinical characteristics, delayed ART initiation after the first ANC visit was not associated with achieving VS at delivery (OR = 0.78; CI = 0.43 to 1.43). This absence of association persisted in an analysis restricted to women with CD4 ≤ 350 cells/μL, who would have been eligible to start ART under either PMTCT guideline (Table S1) and when VS was defined as VL < 1000 copies/mL (Table S2). Moreover, when the analysis was restricted to women initiating ART under Option B+, delayed ART initiation was not associated with VS at delivery (OR = 1.14; CI = 0.61 to 2.16) (Table S3). VL at first ANC visit and duration on ART prior to delivery were significant predictors of VS at delivery in all models, consistent with our previous findings 29. A one‐month increase in time on ART was associated with a 3‐fold increased odds of achieving VS at delivery (OR = 3.14; CI = 1.89 to 5.22). A one‐unit increase in log VL at first ANC visit was associated with a 69% decreased odds of VS at delivery (OR = 0.31; CI = 0.20 to 0.46). In addition, women with a CD4 > 350 cell/μL were twice as likely to have suppressed VL at delivery compared to those with CD4 ≤ 350 cell/μL (OR = 1.92; CI = 0.99 to 3.74), but this association was marginally significant (*p* = 0.054).

**Table 3:** Unadjusted and adjusted logistic regression models of the association between VS (VS < 50 copies/mL) at delivery and timing of ART initiation

	Unadjusted			Adjusted		
	OR	95% CI	*p* value	aOR	95% CI	*p* value
Age in years	1.01	0.96 to 1.05	0.749	1.02	0.97 to 1.08	0.383
Scholar /employed	1.06	0.66 to 1.71	0.481	0.78	0.43 to 1.43	0.429
Gestation (weeks)	0.90	0.87 to 0.93	<0.001	1.11	0.99 to 1.24	0.086
CD4 (cell/μL) 0 to 350	1	(ref)		1	(ref)	
>350	2.03	1.25 to 3.30	0.004	1.92	0.99 to 3.74	0.054
PMTCT Option A	1	(ref)		1	(ref)	
Option B+	0.70	0.37 to 1.31	0.270	0.47	0.18 to 1.24	0.125
Median mths on ART	1.70	1.46 to 1.98	<0.001	3.14	1.89 to 5.22	<0.001
Baseline Viral load	0.38	0.27 to 0.53	<0.001	0.31	0.20 to 0.46	<0.001
Delay to ART initiation						
Delayed (>2 days)	1	(ref)		1	(ref)	
Same‐day (0 to 2 days)	0.71	0.41 to 1.22	0.218	0.78	0.43 to 1.43	0.808

### 3.4 Time to first viral rebound and viral suppression at 12 months postpartum

**Figure 1:**
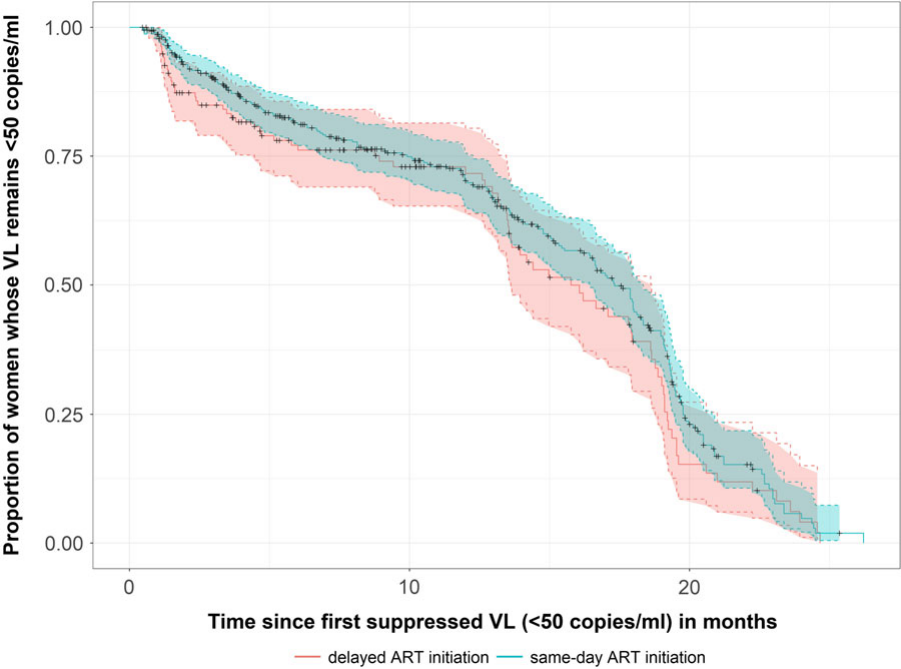
Probability of maintaining viral suppression (VS < 50 copies) following initial suppression and 95% confidence intervals compared according to timing of ART initiation since first ANC visit.

At enrolment, the cohort had a median log VL = 4.00 log_10_ copies/mL (IQR = 3.4 to 4.6) and 4% (n = 25) had VL < 50 copies/mL. At subsequent visits, an additional 527 women achieved VS, with a total of 511(81%) women having at least one return visit following initial VS. The median number of study visits after initial VS was 6 (IQR = 3 to 7), with more follow‐up visits among women starting ART under Option B+ (median = 6; IQR = 3 to 7) *versus* Option A (median = 5, IQR = 3 to 6). In a Cox proportional hazard model adjusting for baseline demographic and clinical characteristics, the rate of viral rebound following initial VS was similar in women who delayed ART initiation compared to those who started ART same‐day (aHR = 0.75; CI = 0.50 to 1.45; Table S4) as shown in Figure 1. Predictors of experiencing viral rebound were shorter duration on ART, earlier gestation and higher VL at first ANC visit (Table S4).

In a subset of women reporting to be breastfeeding at first postpartum visit (n = 471; 65% of the cohort); same‐day = 345 (75%) and delayed = 126 (73%) had a VL test done at 12 m postpartum. Levels of VS between same‐day ART initiators and those who delayed ART start remained comparable, even when VS was defined at VL < 1000 copies/mL or restricted by CD4 cell count at enrolment (Table 4).

**Table 4:** Adjusted association between (1) suppressed viral load and same‐day ART initiation and (2) Engagement in care at 12 months postpartum and same‐day ART initiation

	1. Breastfeeding (n = 471) [aOR (95% CI)]		2. All women (n = 628) [aOR (95% CI)]
	VS < 50	VS < 1000	Engagement
All women	1.33 (0.71 to 2.53)	1.38 (0.71 to 2.71)	1.48 (0.85 to 2.58)
Pre‐ART CD4 (cell/μL) 0 to 350	0.88 (0.35 to 2.20)	1.12 (0.43 to 2.88)	1.21 (0.54 to 2.75)
>350	1.70 (0.65 to 4.47)	1.54 (0.56 to 4.26)	1.61 (0.73 to 3.56)
HIV diagnosis: new in pregnancy	1.52 (0.57 to 4.02)	1.42 (0.53 to 3.79)	1.23 (0.58 to 2.62)
before pregnancy	1.07 (0.45 to 2.58)	1.25 (0.48 to 3.24)	2.03 (0.85 to 4.87)
Age: <28 years	1.20 (0.49 to 2.95)	1.32 (0.54 to 3.26)	1.67 (0.80 to 3.49)
≥28 years	1.56 (0.59 to 4.14)	1.63 (0.56 to 4.71)	1.26 (0.49 to 3.23)

All models adjusted for pre‐ART VL, time on ART, gestation at ART initiation, marital status and PMTCT Option (A/B+); models with all women also adjusted for CD4, age and timing of HIV diagnosis

### 3.5 Engagement in care at 12 months postpartum

All 628 women were followed‐up using routinely collected data to measure engagement in care at 12 months. In this analysis, there were no differences in engagement in care through 12 m postpartum between delayed *versus* same‐day ART initiators (aOR = 1.48; CI = 0.85 to 2.58). This remained the case when analysis was restricted by CD4 cell count, age and HIV diagnosis at first ANC (Table 4).

## 4 Discussion

This analysis suggests that same‐day ART initiation is not associated with worsened or improved maternal outcomes during pregnancy or postpartum. Among pregnant women with delays in ART initiation of up to 4 weeks, VS levels and engagement in care at 12 m postpartum did not differ compared to those initiating ART same‐day. There was no difference in time to first viral rebound by timing of ART initiation. Given the need to maximize the duration on ART prior to delivery and reduce the risk of MTCT, our findings have important implications for the design of services for HIV‐positive pregnant and breastfeeding women.

Few studies have compared maternal outcomes in PMTCT programmes across timing of ART initiation. Our findings of 77% VS at delivery were consistent with observed VS levels at delivery in cohorts of ART‐naïve pregnant women in the United Kingdom (77% VS < 50 copies/mL), Benin (71% VS < 40 copies/mL) and South Africa (76% VS < 50 copies/mL) 30, 31, 32. Engagement in care at 12 m postpartum was consistent with those reported in other Sub‐Saharan settings; 77% among women who started ART under option B+ in Malawi and 81% among women starting ART under both Option A and Option B+ in Cameroon 33, 34, 35.

In this analysis, delay in ART initiation was used as a proxy for the time available for patient preparation before starting ART. Although there is little agreement in the literature comparing the benefits of pre‐therapy counselling to concurrent counselling, our findings support our earlier findings and those from other studies that have indicted no clear benefits in maternal outcomes associated with pre‐therapy counselling 5, 25. Completing adherence counselling prior to ART initiation was not associated with VS at three months among a Ugandan adult cohort initiating ART due to low CD4 cell count 36. In contrast, a randomized control trial in Kenya reported that adult patients who received at least 3 pre‐therapy counselling sessions prior to ART initiation were 59% less likely to experience viral failure at four months post‐ART initiation compared to patients who did not receive pre‐therapy counselling 37. The differing findings in adult populations may be attributed to methodological variation, differing definitions of VS, ART regimens and observation periods.

Two significant predictors for VS were identified in this analysis. A low VL at first ANC visit was associated with increased odds of being virally suppressed at delivery, as was increased time on ART. This is consistent with findings reported in our earlier work and by previous studies assessing levels of adherence and VS in pregnancy 26, 29, 38. In this cohort, patients initiating ART under Option B+ were more likely to be offered same‐day ART initiation compared to women starting ART under Option A, and the independent effect of this difference in programme structure cannot be entirely isolated from this analysis. Despite this, we conducted a sub‐analysis restricted to women with CD4 ≤ 350 cells/μL who would have been eligible to start ART under both PMTCT guidelines, and the lack of association between delayed ART initiation and VS persisted. With each additional week on treatment potentially reducing the risk of perinatal HIV transmission, our data highlights the potential gains in MTCT reduction specifically in developing countries where women commonly present for ANC late in the second trimester 7.

This evidence may have important implications for the implementation of universal ART policies for non‐pregnant adults as recommended by WHO 39. The findings provide preliminary suggestions that HIV‐infected individuals might accept immediate initiation of lifelong treatment and be adherent to ART even when identified as ART‐eligible (and possibly HIV‐infected) on the same day. While evidence from PMTCT services should be extrapolated with caution given the motivation for adherence during pregnancy is often the health of the infant, this possibility is an important avenue for future research.

Several limitations need to be considered when interpreting these findings. Data on the delay to ART initiation was self‐reported by study participants. Although all interviews were conducted by trained staff, separate from routine care, this measure could be susceptible to social desirability bias leading to an overestimation of women reporting same‐day ART initiation and pulling the association towards the null. It is also important to note that the reasons for delayed ART initiation were not recorded, and thus we are unable to separate systematic (related to laboratory results) delays from patient choice. With delays being observed under both PMTCT guidelines, we believe sources of delays were a combination of the two influences, and further research is required to understand how different sources of delays in ART initiation may impact on subsequent adherence, positively or negatively. Delays to ART initiation associated with medical reasons may overestimate the number of women who delayed ART initiation and experiences better outcomes. This study is likely to underestimate engagement in care as we relied on routinely collected data which varies in completeness and quality for both comparison groups. Participation in the parent study may have been likely to positively affect engagement in routine care, but it is not clear how this may differ by timing of ART initiation. In addition, the study design employed could not control for unknown and unmeasured confounders, which may account for the observed null association. Although secondary analyses such as this may have limitations, they also offer the best available information given the urgency of timely ART initiation during pregnancy. Finally, the generalizability of these findings should be treated with caution, as maternal outcomes and patient preparation are likely to be context‐specific, and vary with the health service and patient population.

## 5 Conclusion

In summary, these findings do not support the hypotheses that delaying ART initiation in pregnancy contributes to worsened or improved maternal outcomes. While these results are reassuring for ART programmes implementing immediate ART initiation during pregnancy, further research is required to examine long‐term engagement in care, particularly postpartum.

## Competing interests

The authors have no competing interests to declare.

## Supporting information


**Figure S1**. Distribution of delay in ART initiation since first ANC visit
**Table S1**. Unadjusted and adjusted logistic regression models for achieving viral suppression (defined as VS < 50 copies/mL) at delivery restricted to women with Pre‐ART CD4< 350 cells/μL
**Table S2.** Unadjusted and adjusted logistic regression models for achieving viral suppression at delivery (defined as VL < 1000 copies/mL) at delivery
**Table S3.** Unadjusted and adjusted logistic regression models for achieving viral suppression at delivery (defined as VS < 50 copies/mL) restricted to women initiating ANC under Option B+
**Table S4**. Proportional hazard model predicting time to first elevated viral load following initial suppression
