# Supplementary material for: Same‐day antiretroviral therapy (ART) initiation in pregnancy is not associated with viral suppression or engagement in care: A cohort study
Source: J Int AIDS Soc. 2018 Jun 22;21(6):e25133. doi: 10.1002/jia2.25133 (PMC6016637; doi:10.1002/jia2.25133)
Supplement: Supplementary file 1 — Figure S1. Distribution of delay in ART initiation since first ANC visit Table S1. Unadjusted and adjusted logistic regression models for achieving viral suppression (defined as VS < 50 copies/mL) at delivery restricted to women with Pre‐ART CD4< 350 cells/μL Table S2. Unadjusted and adjusted logistic regression models for achieving viral suppression at delivery (defined as VL < 1000 copies/mL) at delivery Table S3. Unadjusted and adjusted logistic regression models for achieving viral suppression at delivery (defined as VS < 50 copies/mL) restricted to women initiating ANC under Option B+ Table S4. Proportional hazard model predicting time to first elevated viral load following initial suppression [file JIA2-21-e25133-s001.docx]

Supplement Inserts

Figure 1: Distribution of delay in ART initiation since first ANC visit

Table 1: *Unadjusted and adjusted logistic regression models for achieving viral suppression (defined as VS<50 copies/ml) at delivery restricted to women with Pre-ART CD4<350 cells/ul*

|  |  | Unadjusted | | |  | Adjusted | | |
| --- | --- | --- | --- | --- | --- | --- | --- | --- |
|  |  | **OR** | **95 % CI** | **P value** |  | **aOR** | **95% CI** | **P value** |
| Age in years |  | 0.98 | 0.92 – 1.04 | 0.537 |  | 1.02 | 0.94 - 1.09 | 0.666 |
| Scholar /employed |  | 1.73 | 0.93 – 3.24 | 0.086 |  | 0.98 | 0.45 – 2.55 | 0.961 |
| Gestation (weeks) |  | 0.88 | 0.84 – 0.93 | <0.001 |  | 1.05 | 0.89 - 1.24 | 0.522 |
| PMTCT Option A |  | 1 | (ref) |  |  | 1 | (ref) |  |
| Option B+ |  | 0.701 | 0.37 – 1.31 | 0.270 |  | 0.47 | 0.14 - 1.60 | 0.225 |
| Median mths on ART |  | 1.87 | 1.49 – 2.33 | <0.001 |  | 2.56 | 1.23 -5.32 | 0.011 |
| Baseline Viral load |  | 0.35 | 0.21- 0.57 | <0.001 |  | 0.311 | 0.18 - 0.53 | <0.001 |
| Delay to ART initiation |  |  |  |  |  |  |  |  |
| Delayed ( >2 days) |  | 1 | (ref) |  |  | 1 | (ref) |  |
| Same-day ( 0-2 days) |  | 0.53 | 0.28 – 1.02 | 0.057 |  | 0.85 | 0.26 – 2.83 | 0.795 |

Table 2: *Unadjusted and adjusted logistic regression models for achieving viral suppression at delivery (defined as VL<1000 copies/ml) at delivery*

|  |  | Unadjusted | | |  | Adjusted | | |
| --- | --- | --- | --- | --- | --- | --- | --- | --- |
|  |  | **OR** | **95 % CI** | **P value** |  | **aOR** | **95% CI** | **P value** |
| Age in years |  | 1.10 | 1.00 – 1.20 | 0.031 |  | 1.02 | 1.01 - 1.21 | 0.030 |
| Scholar /employed |  | 1.07 | 0.45– 2.51 | 0.870 |  | 1.07 | 0.41 – 2.76 | 0.895 |
| Gestation (weeks) |  | 0.95 | 0.89 – 1.00 | 0.061 |  | 1.14 | 0.97 - 1.33 | 0.109 |
| CD4 (cell/μL) 0 -350 |  | 1 | (ref) |  |  | 1 | (ref) |  |
| >350 |  | 1.04 | 0.46 – 2.39 | 0.919 |  | 0.87 | 0.32 – 2.40 | 0.789 |
| PMTCT Option A |  | 1 | (ref) |  |  | 1 | (ref) |  |
| Option B+ |  | 0.59 | 0.17 – 2.01 | 0.397 |  | 0.41 | 0.09 - 1.97 | 0.268 |
| Median mths on ART |  | 1.34 | 1.09 – 1.76 | 0.007 |  | 2.45 | 1.25 – 4.80 | 0.009 |
| Baseline Viral load |  | 0.45 | 0.26- 0.79 | 0.005 |  | 0.39 | 0.21 - 0.73 | 0.003 |
| Delay to ART initiation |  |  |  |  |  |  |  |  |
| Delayed ( >2 days) |  | 1 | (ref) |  |  | 1 | (ref) |  |
| Same-day ( 0-2 days) |  | 0.89 | 0.34 – 2.32 | 0.825 |  | 1.05 | 0.25 – 4.44 | 0.167 |

Table 3: *Unadjusted and adjusted logistic regression models for achieving viral suppression at delivery (defined as VS<50 copies/ml) restricted to women initiating ANC under Option B+*

|  |  | Unadjusted | | |  | Adjusted | | |
| --- | --- | --- | --- | --- | --- | --- | --- | --- |
|  |  | **OR** | **95 % CI** | **P value** |  | **aOR** | **95% CI** | **P value** |
| Age in years |  | 1.00 | 0.96 – 1.05 | 0.857 |  | 1.02 | 0.97 - 1.08 | 0.433 |
| Scholar /employed |  | 1.16 | 0.68 – 1.96 | 0.582 |  | 0.87 | 0.45 - 1.68 | 0.683 |
| Gestation (weeks) |  | 0.91 | 0.88 – 0.94 | <0.001 |  | 1.12 | 0.98 - 1.28 | 0.084 |
| CD4 (cell/μL) 0 -350 |  | 1 | (ref) |  |  | 1 | (ref) |  |
| >350 |  | 2.26 | 134 – 3.82 | 0.002 |  | 1.96 | 0.84 – 3.40 | 0.139 |
| Median mths on ART |  | 1.60 | 1.36 – 1.87 | <0.001 |  | 3.07 | 1.74 – 5.45 | <0.001 |
| Baseline Viral load |  | 0.33 | 0.22 – 0.49 | <0.001 |  | 0.29 | 0.18 - 0.45 | <0.001 |
| Delay to ART initiation |  |  |  |  |  |  |  |  |
| Delayed ( >2 days) |  | 1 | (ref) |  |  | 1 | (ref) |  |
| Same-day ( 0-2 days) |  | 0.76 | 0.35 – 1.66 | 0.499 |  | 0.73 | 0.24 – 2.23 | 0.578 |

Table 4*:  Proportional hazard model predicting time to first elevated viral load following initial suppression*

| OVERALL |  | Unadjusted | | |  | Adjusted | | |
| --- | --- | --- | --- | --- | --- | --- | --- | --- |
|  |  | **HR** | **95 % CI** | **P value** |  | **aHR** | **95% CI** | **P value** |
| Age in years |  | 0.97 | 0.94 – 1.01 | 0.091 |  | 0.96 | 0.93 – 0.99 | 0.006 |
| Formal housing |  | 1.31 | 0.92 – 1.84 | 0.130 |  | 0.85 | 0.59 - 1.24 | 0.400 |
| Gestation (weeks) |  | 0.900 | 0.87 – 0.93 | <0.001 |  | 1.03 | 1.01-1.05 | 0.004 |
| CD4 (cell/μL) 0 -350 |  | 1 | (ref) |  |  | 1 | (ref) |  |
| >350 |  | 1.01 | 0.71 – 1.43 | 0.969 |  | 0.69 | 0.52 – 0.93 | 0.013 |
| PMTCT Option A |  | 1 | (ref) |  |  | 1 | (ref) |  |
| Option B+ |  | 0.87 | 0.57 – 1.34 | 0.539 |  | 1.12 | 0.73 - 1.72 | 0.227 |
| Baseline Viral load |  | 0.38 | 0.27- 0.53 | <0.001 |  | 2.25 | 1.73- 2.86 | <0.001 |
| Delay to ART initiation |  |  |  |  |  |  |  |  |
| Delayed ( >2 days) |  | 1 | (ref) |  |  | 1 | (ref) |  |
| Same-day ( 0-2 days) |  | 0.95 | 0.46 – 1.41 | 0.798 |  | 0.75 | 0.50 -1.45 | 0.069 |
